# Mobilisation of distributional data for vascular plants of Murmansk Region, Russia: Digital representation of the *Flora of Murmansk Region*

**DOI:** 10.3897/BDJ.8.e59456

**Published:** 2020-11-18

**Authors:** Mikhail N. Kozhin, Sampsa Lommi, Alexander N. Sennikov

**Affiliations:** 1 Avrorin Polar-Alpine Botanical Garden-Institute, Apatity, Russia Avrorin Polar-Alpine Botanical Garden-Institute Apatity Russia; 2 Lomonosov Moscow State University, Moscow, Russia Lomonosov Moscow State University Moscow Russia; 3 University of Helsinki, Helsinki, Finland University of Helsinki Helsinki Finland; 4 Komarov Botanical Institute, Saint Petersburg, Russia Komarov Botanical Institute Saint Petersburg Russia

**Keywords:** angiosperms, ferns, gymnosperms, Kola Peninsula, lycophytes, mapping, plant distribution, Russian Lapland

## Abstract

**Background:**

The present-day demand for digital availability of distributional data in biodiversity studies requires a special effort in assembling and editing the data otherwise scattered in paper literature and herbarium collections, which can be poorly accessible or little understood to present-day users and especially automatic data processors. Although the vascular plants of Murmansk Region (northern part of European Russia) are well studied and represented in publications, the accessibility of this knowledge is highly insufficient. The most widely known source is the *Flora of Murmansk Region* (published in 1953–1966), which remains in use because of its high original quality, detailed elaboration and completeness. We consider digitising this source to be of primary importance in biodiversity studies in the Arctic Region because of its point occurrence maps, which were based on the comprehensive inventory of contemporary herbarium collections.

**New information:**

We have compiled a dataset based on 554 printed point occurrence maps of species distributions published in the *Flora of Murmansk Region*, which includes 25,555 records of georeferenced plant occurrences that belong to 1,073 species and 5 hybrids. The occurrences are ultimately based on herbarium specimens kept at KPABG and LE, which were collected during 1837–1965. We estimate that these specimens represent ca. 60% of the current global herbarium holdings originated from Murmansk Region; this means that the dataset gives a fair representation of the regional flora.

## Introduction

**Figure 1.**
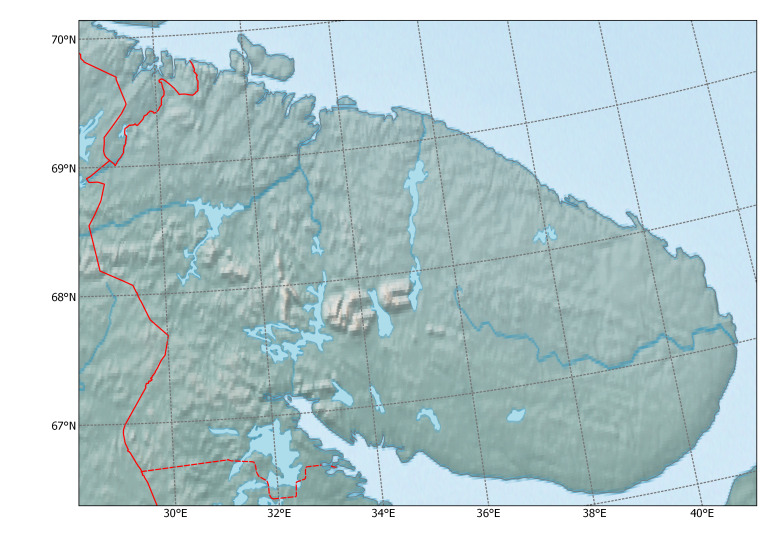
Topography of Murmansk Region.

Murmansk Region is a northern administrative territory in European Russia, which includes parts of two historical provinces: a large part of Lapland (represented by tundra and forest tundra) and northern Karelia (represented by northern boreal forest). This extensive territory (Fig. 1) lies mostly above the Polar Circle and is therefore considered part of the Arctic ecosystems.

The flora of vascular plants of Murmansk Region has been actively studied for 200 years and, therefore, the Region is among the best researched botanical territories in Russia. This situation is reflected in the *Flora of Murmansk Region* (Gorodkov 1953, Poyarkova 1954, Poyarkova 1956, Poyarkova 1959, Poyarkova 1966), which remains among the best floristic inventories in Russian administrative territories. Besides this synoptic work, there is a large corpus of other botanical publications which are based on many thousands of herbarium specimens.

Despite the good state of the botanical knowledge on Murmansk Region in general, there are some significant shortcomings hindering its use. One is a complicated history of studies, which resulted in the splitting of efforts and the dispersal of herbarium collections. The flora of this territory was independently studied by Russian and Finnish botanists, who accumulated a vast knowledge that remains separate.

The Finnish botanical studies in the Kola Peninsula started with the private study of J. Fellman (Väre 2011), who compiled the first scientific checklist (332 species) on the basis of his collections and observations (Fellman 1831). The mid-19^th^ century inventories recorded 517 species of vascular plants (Nylander and Sælan 1859). The second checklist (517 species) and the pioneering vegetation study (Fellman 1869) was also Finnish, made by N.I. Fellman on the basis of expeditions organised by *Societas pro Fauna et Flora Fennica* in 1861 and 1863 (Sennikov and Kozhin 2018, Kozhin and Sennikov 2020). It was followed by the Great Kola Expedition in 1887, which aimed at exploring the features of geography, geology, vegetation and flora of the Kola Peninsula (Uotila 2013). The resulting inventory (Sælan et al. 1889) listed 565 species (*Hieracium* excluded) in Russian Lapland. In the 20^th^ century, Finnish botanists remained active in the Kola Peninsula (Uotila 2013); Finnish botanical records from this territory were incorporated in the largest Finnish floristic monographs (Cajander 1906), with the final figure of 576 species of vascular plants recorded.

The Finnish herbarium collections from present-day Murmansk Region were deposited at the Botanical Museum, University of Helsinki (H). These collections were inventoried by Hjelt (Hjelt 1888, Hjelt 1892, Hjelt 1895, Hjelt 1902, Hjelt 1906, Hjelt 1911, Hjelt 1919, Hjelt 1923, Hjelt 1926).

The Russian botanical exploration of the Kola Peninsula started very early, with the pioneering observations made during the Russian academic expeditions of 1768–1774 (Sennikov and Kozhin 2018), but the first inventory appeared only in *Flora Rossica* (Ledebour 1841, Ledebour 1843, Ledebour 1847, Ledebour 1852). The next significant study, supported by the Saint-Petersburg Society of Naturalists, was the geobotanical exploration by K. Regel in 1913. Since 1917, the number of Russian academic expeditions has greatly increased and ultimately resulted in publication of the *Flora of Murmansk Region* (Gorodkov 1953, Poyarkova 1954, Poyarkova 1956, Poyarkova 1959, Poyarkova 1966).

The Russian botanical collections were deposited mostly at the Komarov Botanical Institute (LE) and the Polar-Alpine Botanical Garden-Institute (KPABG).

These two streams of the botanical activity in Murmansk Region have always been separate. The resulting publications were taken into account by the other research side to a limited extent, and the collections have been kept and examined separately. This situation affected and handicapped all major synopses on the flora of Murmansk Region that appeared to date.

The second shortcoming of the Murmansk botanical data is its poor accessibility according to modern standards. There is no common bibliography and index for the published literature, and herbarium collections are divided between towns and countries and not databased.

Since 2016, a joint team of botanists of the Moscow State University and the University of Helsinki undertook a complete and detailed inventory of the flora of Murmansk Region, in order to bring together the Finnish and Russian data on a modern basis. Part of this effort is data inventory and mobilisation.

In the present contribution, we aim to mobilise the distributional data on vascular plants published in the *Flora of Murmansk Region*, which is the greatest botanical dataset from the territory that has ever been compiled. Its value rests on its complete coverage, both taxonomic and territorial, but also on the precision and quality of data collection which remains largely unsurpassed.

Due to the complexity of the original data and the significantly long timeframe of its production, certain insights into the history of the data collection and compilation is needed in order to make potential users better understand the structure and limitations of the dataset. For this reason, we provide a brief description of the data structure in connection with its history, as part of the documentation accompanying the dataset.

## General description

### Purpose

The present project aimed at digitising the data on distribution of vascular plants in Murmansk Region, Russia, which were published as printed point occurrence maps in the *Flora of Murmansk Region* (Gorodkov 1953, Poyarkova 1954, Poyarkova 1956, Poyarkova 1959, Poyarkova 1966).

### Additional information

**History of preparation, structure, data origin**: *Flora of Murmansk Region* became the main scientific task for the staff of the Polar-Alpine Botanical Garden-Institute (Kuzeneva 1963, Shlyakov 1968). The work was initiated immediately after the Second World War, in 1946. The project was originally supervised by Prof. B.N. Gorodkov (1890–1953), who died shortly after the first volume of the *Flora* had been prepared. His successor was A.I. Poyarkova, an active and experience taxonomist from the Komarov Botanical Institute, with an interest in critical groups of vascular plants. The project leader at Kirovsk was O.I. Kuzeneva. Thirteen botanists took part in taxonomic treatments. In Kirovsk, major treatments were prepared by O.I. Kuzeneva, N.I. Orlova, N.Z. Semenova-Tian-Shanskaya, E.G. Chernov and R.N. Shlyakov, and smaller treatments by E.V. Shlyakova and N.A. Avrorin. Experts from outside were involved from the Komarov Botanical Institute (B.N. Gorodkov, A.I. Poyarkova, E.A. Selivanova-Gorodkova, I.A. Linchevsky and S.V. Yuzepchuk), the Leningrad Pedagogical University (V.V. Pisiyaukova) and the Institute of Botany in Kiev (M.V. Klokov).

The treatments written by Kuzeneva included taxonomically difficult groups of plants (several genera of Poaceae, *Carex*, Fabaceae, Caryophyllaceae, Rubiaceae etc.); these treatments are also most detailed and technically accurate. Shlyakov revised other most difficult groups, including Juncaceae, Salicaceae and *Hieracium*. His treatment of *Salix* was accepted in subsequent authoritative monographs (Skvortsov 1968). Shlyakov’s revision of *Hieracium* in Murmansk Region was a taxonomic monograph itself, including 83 apomictic species new to science and dozens of new records; this treatment became the basis for subsequent revision of the genus in East Europe (Shlyakov 1989). Several novelties were introduced in the treatments by Orlova (Poyarkova 1956, Poyarkova 1966), who described four new species in *Betula*, *Alnus*, *Achillea* and *Sonchus* (mostly rejected in later revisions).

The first estimations stated that the flora of Murmansk Region probably includes 700 species of vascular plants (Gorodkov 1953); this figure was corrected to 1160 species when the *Flora* was completed (Poyarkova 1966).

The structure of the *Flora* is traditional; it includes the main features of nomenclature, morphological descriptions, ecological data, distributional data and casual comments. The layout of the work was followed consistently from the first to the last volume of the *Flora* by all its contributors (Poyarkova 1954, Kuzeneva 1963).

The nomenclature is limited to accepted names and main synonyms, with references to protologues, but excluding typifications. Standard references include *Flora Rossica* (Ledebour 1841, Ledebour 1843, Ledebour 1847, Ledebour 1852), *Flora of the USSR* (Bobrov 1965), Perfiliev (1934), Perfiliev (1936), Cajander (1906), Holmberg (1922) for volume 1 only, Holmberg (1926), Holmberg (1931)f or volumes 1–3, Lindman (1926) and Hultén (1950) for volumes 2–5, Mishkin (1953) for volumes 3–5 and Hiitonen (1933) for volumes 4–5.

The information on the presence of a certain species in the territory was based largely on examination of herbarium specimens, except for some records derived solely from published sources (e.g. Hultén 1950) when foreign collections were inaccessible. Some species had been provisionally included in anticipation of actual records in the future; such records (e.g. *Pteridium aquilinum, Botrychium lanceolatum* and *Stratiotes aloides*) were largely confirmed later.

Data on ecology were derived from herbarium specimens and personal observations. Distribution areas were derived from references. Economic importance and use were mentioned when available. Illustrations were an important part of the work. Original drawings (complete species plates, main drawings with separate details) were made mostly by N.Z. Semenova-Tian-Shanskaya (378 plates); after her death, the work was finished by A.V. Dombrovskaya (55 plates) and T.N. Shishlova (4 plates).

The original idea was proposed to include lists of specimens examined for each species (standardised to cite: locality, time, collector). This idea was found unrealistic, and ultimately the distributional data were limited to verbal characteristics and maps (Kuzeneva 1963).

**Figure 2.**
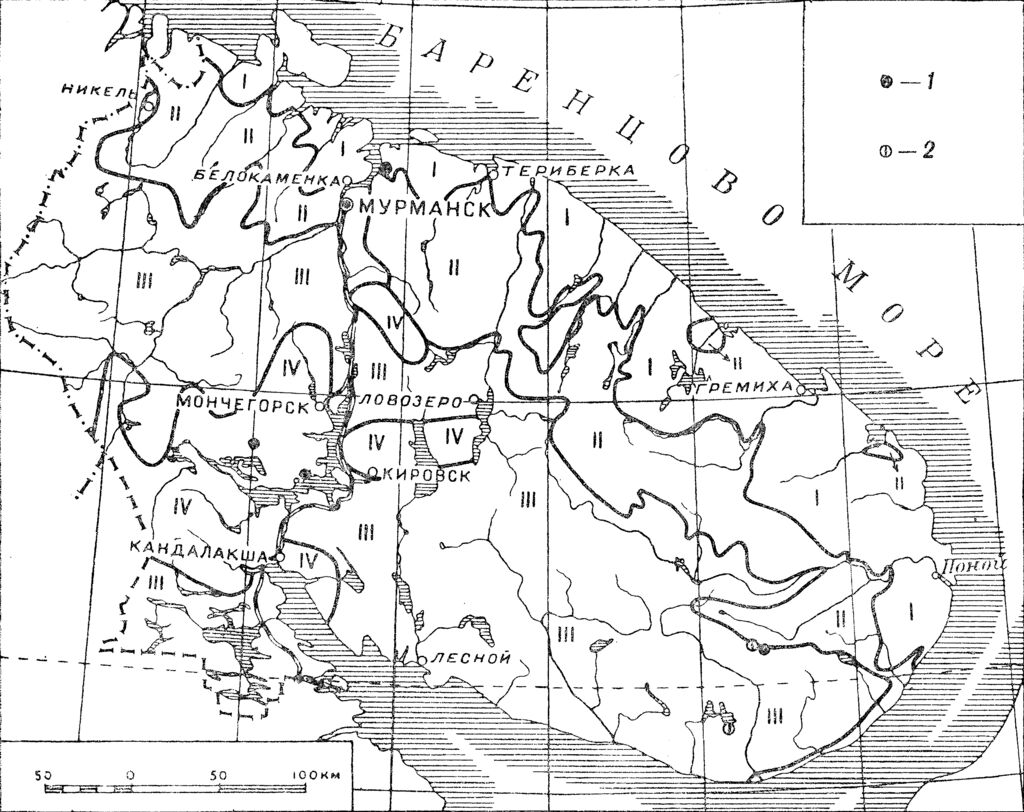
Distribution map of *Isoetes lacustris* (1) and *I. echinospora* (2) (Gorodkov 1953), showing the territorial limits of the *Flora* and the geobotanical districts as accepted in volumes 1–3.

**Figure 3.**
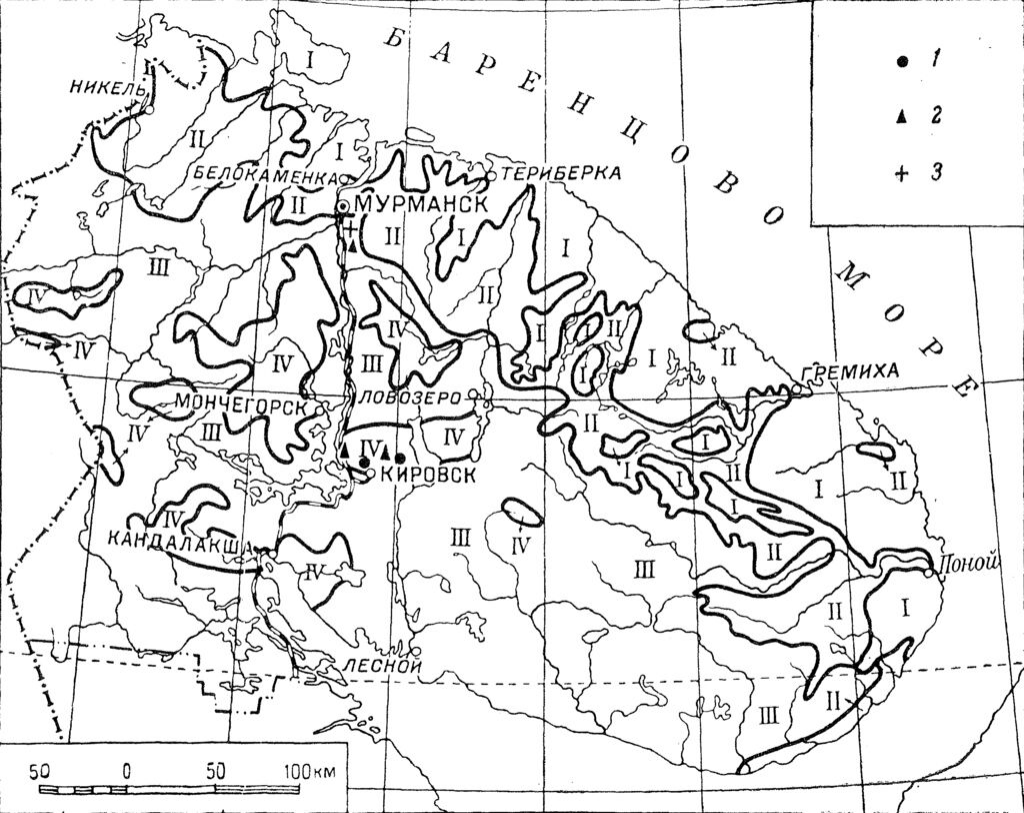
Distribution map of *Alchemilla glabricaulis* (1), *A. micans* (2) and *A. semilunaris* (3) (Poyarkova 1959), showing the territorial limits of the *Flora* and the geobotanical districts as accepted in volumes 4–5.

Maps are a very important part of the *Flora*; at that time, point occurrence distribution maps were extremely uncommon in regional treatments. As a rule, maps are provided for each species treated; the maps were compiled exclusively by E.G. Chernov, who had an extensive field experience in Murmansk Region and, at the same time, worked on the vegetation map of the territory (Chernov 1956, Chernov 1971). Generalised features of the vegetation map were treated as geobotanical districts (Gorodkov 1953: 5), whose limits were shown on each plant distribution map. Four vegetation types (districts) were delimited: tundra, forest tundra, sparse forest and oroarctic zone. This scheme evolved when the *Flora* proceeded: in volumes 1–3, the delimitation was rather coarse (Fig. 2), but in volumes 4–5, it became more detailed and realistic (Fig. 3).

Plant occurrences on the distribution maps were indicated by points; the points were based on herbarium specimens identified or seen by the authors of the corresponding taxonomic treatments. Due to this strict policy, distributional data published in Hultén (1950) were taken into account in treatments but never shown on maps. Herbarium records for 176 widely distributed species were complemented by hatched contours denoting the areas where the species were deemed common (hatch scale implies differences in frequency); those data were derived by E.G. Chernov from his vegetation map. Besides, 120 species were considered common and covering the whole territory, which was therefore completely hatched for those species. Altogether, the *Flora* contains 554 maps with distributional data for 1,073 species and 5 hybrids.

**Original data collection**: The *Flora* was largely based on herbarium collections, which were complemented by field observations for common plants. At that time, the main collections originated from Murmansk Region were deposited at the Komarov Botanical Institute (LE) and the Polar-Alpine Botanical Garden (KPABG).

The collection of the Komarov Botanical Institute was the largest at the time. Based on example groups, we estimate the number of its Murmansk specimens available to the authors of the *Flora* at about 35,000. This collection was established mostly by expeditions of the Russian Academy of Sciences, starting from the Russian Arctic expedition in 1837 under the command of Karl E. von Baer (Baer 1837a, Baer 1837b, Sennikov and Kozhin 2018). The first large acquisition was received from A. Schrenk who travelled in Russian Lapland in 1839; his specimens were made available to C.F. von Ledebour and became the basis of his data from the territory in *Flora Rossica* (Ledebour 1841, Ledebour 1843, Ledebour 1847, Ledebour 1852). Another milestone was the expeditions of K. Regel in 1911–1913, whose outputs were summarised in a series of monographs (Regel 1927, Regel 1928, Regel 1923). After 1917, the territory was actively explored by several large academic expeditions, which resulted also in a large amount of botanical collections deposited at LE. Most notably, Yu.D. Zinserling travelled and collected in Khibiny Mts. (1925) and the eastern parts of the territory (1927–1928), whose vegetation he subsequently described (Zinserling 1929, Zinserling 1934, Zinserling 1935).

Besides the specimens collected by Russian collectors, the Herbarium of the Komarov Botanical Institute (LE) possessed important exsiccata from early Finnish collectors, N.I. Fellman’s *Plantæ Arcticæ Exsiccatæ* distributed by the author through the Botanical Museum, University of Helsinki (Fellman 1869, Sennikov and Kozhin 2018) and F. Nylander’s specimens included in E.M. Fries’ *Herbarium normale plantarum rariorum et criticarum Sueciae* and distributed by the author through booksellers in Uppsala, Sweden (Fries 1837–1865, Väre 2007, Väre 2008). Despite the small number of specimens (Fellman: 360 specimens, Nylander: about 10 specimens), these collections were selected as representative samples of the whole flora, distributed to major Herbaria in various countries and widely consulted and cited in botanical publications. Besides the exsiccata, a set of duplicates collected by Finnish botanists (V. Brotherus, N.I. Fellman, M. Brenner, O. Kihlman) was received by LE from H before 1917.

Another large collection from Murmansk Region, which included a large number of specimens from historical and many recent expeditions, is kept at the Botanical Museum, University of Helsinki (Uotila 2013). The size of this collection is estimated at 25,000 specimens; about 2,000 specimens were distributed as duplicates in other Finnish Herbaria: Åbo Akademi University (TURA, now transferred to TUR), University of Oulu (OULU), University of Turku (TUR) etc. This collection was established in the 19^th^ century with expeditions organised by the *Societas pro Fauna et Flora Fennica* (summary of the holdings was published in Sælan et al. 1889) and much complemented with numerous expeditions and excursions to the north-western and south-western parts of present-day Murmansk Region in the first half of the 20^th^ century (Uotila 2013). During the preparation of the *Flora*, these collections were taken into account to a very minor extent (mostly the exsiccata and duplicate specimens from H, which were deposited at LE, and a few records derived from the Finnish botanical literature). The only exception was the treatment of *Hieracium* (Poyarkova 1966), for which some type specimens were requested on loan from H, S and UPS.

When the *Flora* was started, the young collections of the Polar-Alpine Botanical Garden were considered minor and complementary. When established, this Herbarium originally included collections from the Lapland Strict Nature Reserve, the Northern Research and Trade Expedition (now Arctic and Antarctic Research Institute), and early expeditions of the Academy of Sciences of the USSR to the Kola Peninsula. According to the first inventory, it numbered 2,000 specimens in 1934. Subsequently, the Herbarium received a set of specimens collected in botanical expeditions to the Kola Peninsula and also a number of duplicates transferred from LE and LECB. Since the local collections in Murmansk Region were very small and the Herbarium of the Komarov Botanical Institute was largely taxonomy-oriented and did not provide a proper coverage of the territory for understanding plant distributions in detail, the new floristic inventory required a massive effort to sample plants in less studied parts of the territory. Prior to the preparation of printed books, the Polar-Alpine Botanical Garden organised numerous expeditions, which continued during the whole period of the preparation. The expeditions thoroughly covered the western part of Lake Imandra with the Tuloma River basin, the northwesternmost parts of the territory (Pechenga District, which was ceded to the USSR by Finland in 1944), the Voronya River basin from Lovozero to Gavrilovo, Lovozero Mts. and the western part of the Lovozero Lake basin, basins of several other large rivers (Varzuga, Strelna, Ponoy), the Kola Bay, a large part of Tersky Coast etc. When the northern part of the former Finnish Kuusamo District was transferred from Karelian ASSR to Murmansk Region in 1955, this territory was visited by special expeditions in 1956–1957 to fill the resulting gap in the botanical information. Among the most frequent participants and active collectors in these expeditions were O.I. Kuzeneva, E.G. Chernov, N.I. Orlova, R.N. Shlyakov, N.A. Avrorin and N.Z. Semenova-Tian-Shanskaya (scientists) and L.R. Ponomareva (preparator). A.I. Poyarkova, the future editor and supervisor of the project, also collected many specimens which, however, were deposited at LE. Some other persons, who revised particular taxonomic groups for the *Flora*, travelled to more accessible areas for smaller collections (Kuzeneva 1963, Shlyakov 1968).

As a result of this effort, the available collections had been rapidly increasing. After the work on the *Flora* had started, the number of specimens at KPABG reached 12,000 in 1950, whereas by the end of this work, in mid-1960s, it exceeded 40,000. This pace implies that due to the work on the *Flora*, the amount of collections at KPABG increased more than 20 times from the original figure of 2,000 (Kuzeneva 1963).

In 1949, the Herbarium of Kandalaksha Strict Nature Reserve was established and acquired specimens from the White and Barents sea coasts. By the end of 1960s, the collection consisted of 2,000 specimens, many of which have been studied by *Flora*'s authors.

Altogether, according to our estimations, over 75,000 herbarium specimens (from LE and KPABG and, to a minor extent, from H) were used in preparation of the *Flora*. It was a nearly complete coverage of collections available in the USSR (except for the Herbarium of the Leningrad State University, LECB and Moscow State University, MW); the foreign collections understandably were not covered because of political restrictions and financial limitations of the times. Among the Russian collections, LECB was excluded with the historical collections of K. Regel and R.F. Nyman (Bubyreva 2013) and MW was omitted with the collections of M.I. Nazarov and N.S.Parfentyeva (Bagdasarova 2006); nevertheless, these omissions hardly caused any significant loss of information.

**Mapped records vs. present-day knowledge**: Since the *Flora* had been completed, the amount of collections changed in the following way. The holdings of KPABG continued growing (although less actively) until 1990s. Some specimens have been added recently to H, which resulted from joint Finnish-Russian expeditions in post-Soviet times (Uotila 2013, Väre 2017). The collections of LE increased mostly due to a large transfer of duplicates from KPABG; besides the duplicates of ordinary specimens, the whole set of holotypes was transferred from KPABG to LE in the 1980s. The amount of unique additions to LE is estimated at 10% of the former holdings. The collections of LECB and OULU increased insignificantly, while the collections of MW and KAND have grown more than five times.

At present, we have the following estimations of the amount of herbarium specimens collected from Murmansk Region and kept in public collections (holdings exceeding 1,000 specimens): KPABG – 45,000, LE – 40,000, H – 25,000, MW – 15,000, KAND – 10,000, LECB – 3,000, OULU – 1,500 and TUR – 1,200. This means that the coverage of the *Flora* dataset (duplicates excluded) is ca. 60% of the present-day herbarium holdings available from the territory.

## Sampling methods

### Sampling description

A total of 554 maps published in volumes 1–5 of the *Flora of Murmansk Region* (Gorodkov 1953, Poyarkova 1954, Poyarkova 1956, Poyarkova 1959, Poyarkova 1966) was scanned and cropped to cover exactly the same area. The scanned maps were processed in R Software Environment (R Core Team 2020) using ‘sp’ package (https://cran.r-project.org/web/packages/sp/index.html).

The printed map projection was determined by the method of trial and error to Lambert Conformal Conic Projection using standard parallels at 68°N and 70°N and central meridian at 36°E. Maps were georeferenced using corners as control points. Corner points were determined separately for two sets of maps using a different design (basemap) in volumes 1–3 (x min: -311000, x max: 278000, y min: -252000, y max: 232000) vs. volumes 4–5 (x min: -311000, x max: 278000, y min: -234000, y max: 230000).

**Figure 4.**
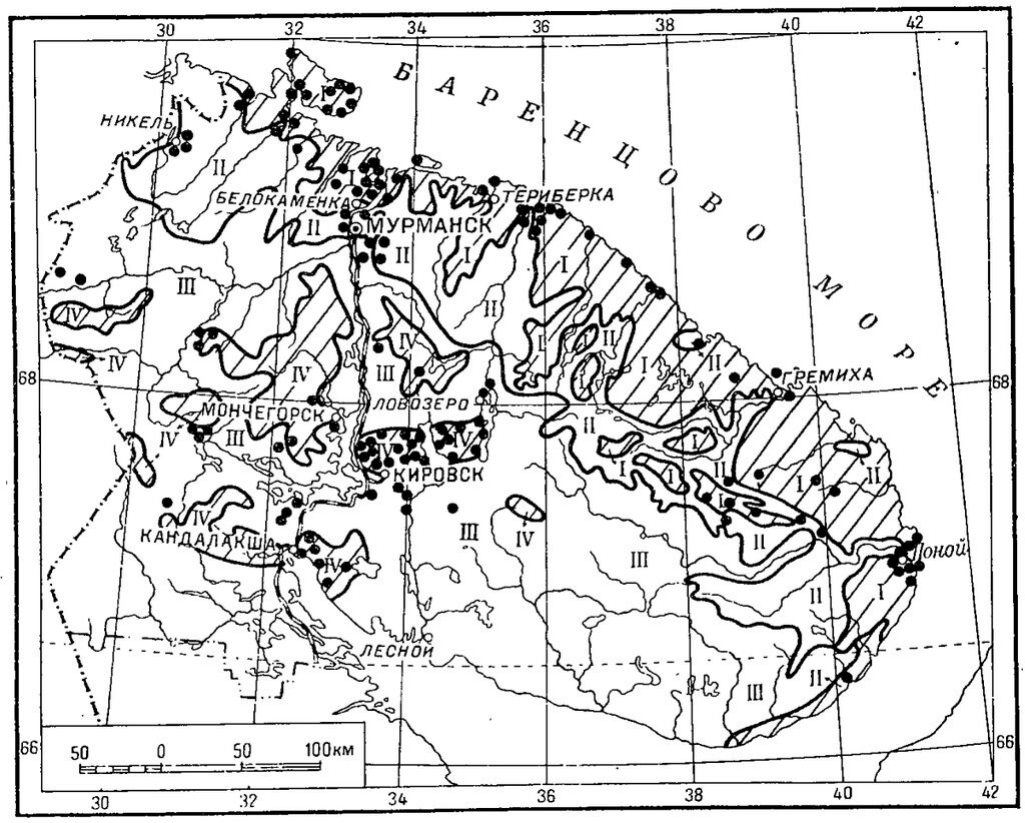
Distribution map of *Phyllodoce caerulea*, original printed map (Poyarkova 1959).

**Figure 5.**
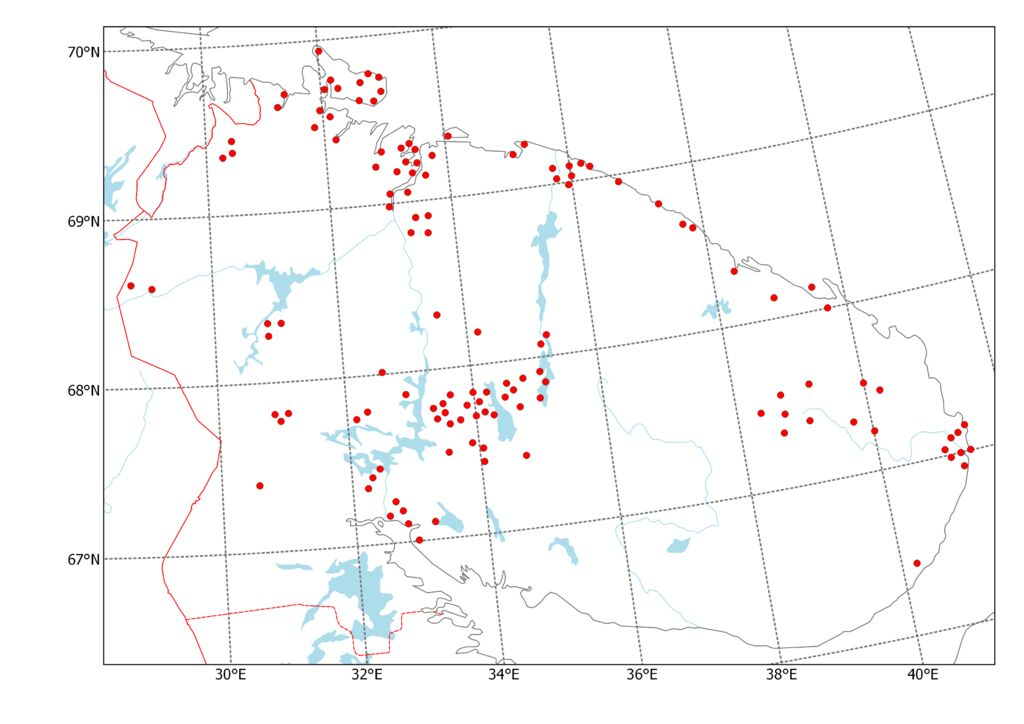
Distribution map of *Phyllodoce caerulea*, digitally recreated from the printed map.

After calibrating the maps, plant record symbols were digitised one by one by mouse clicking using the WGS 84. Positions of records in some areas were slightly adjusted manually to match the landscape features when the printed base map was found distorted. As an example of this work, one original map (Fig. 4) and its corresponding digital map (Fig. 5) are included here.

Coordinate uncertainty was established considering the size of symbols, the accuracy of printed basemaps and the precision of old herbarium labels used for mapping. The level of accuracy was estimated at 5 km and used throughout the dataset.

**Figure 6.**
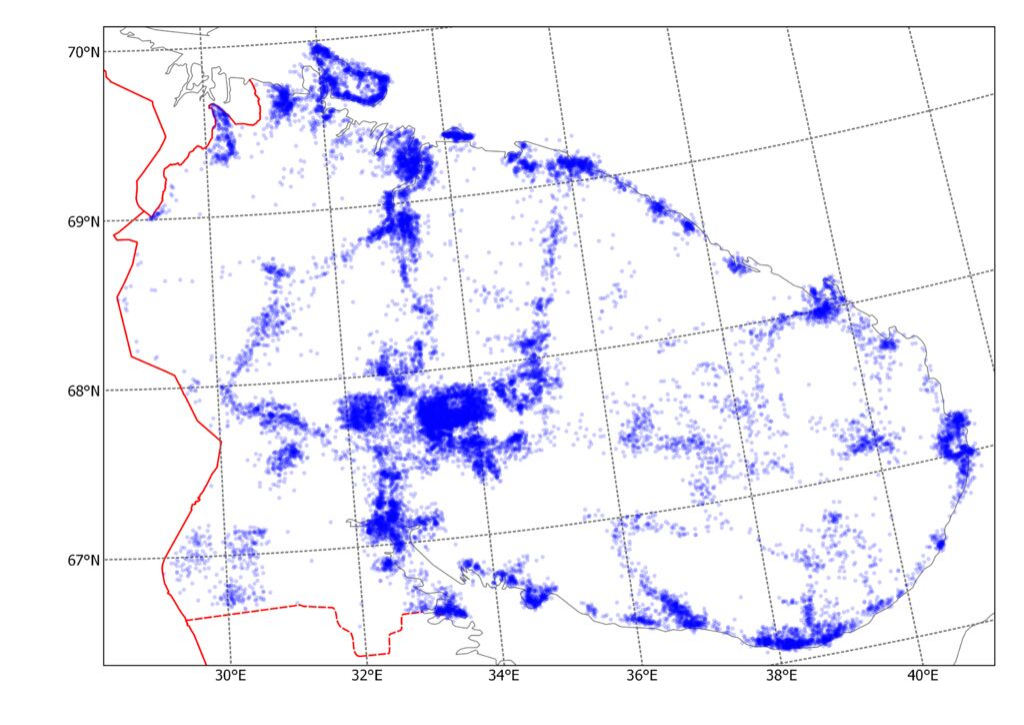
Summary map of all records extracted from the *Flora of Murmansk Region*.

Altogether, 25,555 records of plant occurrences have been extracted from the printed maps and databased (Kozhin et al. 2020, Suppl. material 1). The records unevenly cover the whole territory of Murmansk Region (Fig. 6). The most densely sampled areas are Khibiny Mts., Lapland State Reserve, Kandalaksha, Murmansk and Kola, Kildin and Dalniye Zelentsy, Pechenga–Liinahamari and Rybachiy Peninsula, Umba, Kovda, Varzuga, Chavanga–Pyalitsa, Sosnovka, Ponoy and Svyatoi Nos. These localities have been known as local biodiversity hotspots or otherwise traditionally visited because of accessibility.

This dataset was incorporated into the database of the project Flora of Russian Lapland (www.laplandflora.ru), which is maintained at the Moscow State University and uploaded to the Finnish Biodiversity Information Facility (FinBIF) (www.laji.fi) at the University of Helsinki.

## Geographic coverage

### Description

**Natural conditions and changing borders**

The study area includes the territory of Murmansk Region of Russia, as delimited at the time when the *Flora* was being produced. Since the Region had been established in 1938, by merging Murmansk Area of Leningrad Region with Kandalaksha District of the Karelian ASSR, its limits expanded; this process also affected the territorial scope of the *Flora* during its preparation. Originally the *Flora* covered the territory, which included areas ceded by Finland to the USSR in 1940 and 1944 (Pechenga District) and also the territory of Jäniskoski-Niskakoski, which was exchanged with the USSR in 1947. These limits were used in volumes 1–2 of the *Flora*. The northern part of Salla District, ceded by Finland to the USSR in 1940, was transferred from the Karelian ASSR to Murmansk Region in 1953 and 1955; this transfer affected the territorial scope of the *Flora* and was reflected in its volumes 3–5.

As of 1955 and nowadays, the territory of Murmansk Region totals 144,900 km^2^ and is largely situated in the Kola Peninsula, bordering Norway, Finland and the Karelian Republic of Russia. The territory is bounded by the Barents Sea in the north and the White Sea in the south and east. It is fully situated within the Fennoscandian Shield, which is composed mostly of gneisses, granites and quartzites with nearly no limestone; bedrocks often being exposed along the sea shore. The territory is largely flat except for two small mountain massifs in the central part (Khibiny, Lovozero) and a few minor uplands in the western and south-western parts (Chuna-Tundra, Kandalaksha etc.). Rivers and lakes are abundant, the Ponoy River and Lake Imandra being the most significant examples. Islands are many along the shoreline.

This territory lies almost completely north of the Arctic Circle, and its climate is mostly subarctic with a minor influence of the polar climate along the northern coast and in the northern islands (Peel et al. 2007). Phytogeographic oceanity is considered higher along the northern coast, lower along the southern coast and lowermost in the mountains (Jäger 1968). The territory is divided between two biogeographic regions, arctic and boreal (Cervellini 2020). The tundra zone is represented as a narrow belt under the arctic climate with dwarf-shrub and dwarf-shrub and lichen communities. Further south follows a belt of forest tundra, with sparse birch woodland, and the south-western part of the territory is occupied by the northern taiga zone represented by forests with a variable dominance of spruce, pine and birch (Gribova et al. 1980).

Present-day permanent human population is about 800,000 people, living in 16 cities and towns and over 100 villages. This area is a native territory of the Saami people, who are indigenous to the Arctic, and the Russian Pomor people who are its long-term residents, and is also home to many people resettled from other parts of Russia, largely in the 19^th^ and 20^th^ centuries in the course of the economic development of the territory. The 20^th^ century was remarkable in the intensely growing level of urbanisation, mining, road construction, maritime transport and military activities, which led to a huge increase in the proportion of alien plants in the flora (Kozhin and Sennikov 2018).

### Coordinates

66.057 and 69.951 Latitude; 28.416 and 41.411 Longitude.

## Taxonomic coverage

### Description

The dataset covers all taxonomic groups traditionally treated as vascular plants, i.e. Lycopodiophyta, Pteridophyta (incl. Pteridopsida and Equisetopsida) and Spermatophytes (incl. Magnoliophyta and Pinophyta), which were recorded and mapped as occurring in Murmansk Region in the *Flora of Murmansk Region* (Gorodkov 1953, Poyarkova 1954, Poyarkova 1956, Poyarkova 1959, Poyarkova 1966). A total of 1073 species and 5 notospecies are included in the dataset. Taxonomic circumscription (species concept and synonymy) and nomenclature (binary names) are original, as used in the *Flora*. Accepted names applied to the mapped taxa are verified and corrections and additions published in volumes 3 and 5 of the *Flora* are taken into account (except for the pair *Leucorchis albidus* and *Platanthera bifolia*, which were mistakenly corrected after the original publication). Misprints and other purely technical errors or formatting are corrected; swapped plant names (misplaced on legends to the printed maps) are applied to correct taxa. For hybrids denoted by formulas in the *Flora*, we added binary combinations to facilitate machine reading.

**Taxonomic concept**: As many other synoptic publications of the time, the *Flora* used the same taxonomic concept as employed in the *Flora of the USSR* (Bobrov 1965). This means species is the main and, in fact, the only widely used rank for accepted taxa, and geographical or ecological variants are formally treated as species (Kuzeneva 1963). This makes the *Flora* data partly incompatible with present-day international practice, requiring an effort to produce a consensus synonymy.

The *Flora* was a critical taxonomic revision, not only an inventory of collections. Some treatments resulted in re-definition of species limits or in establishing new taxa. Many revisions, especially with taxonomic novelties, were published separately as background data. Examples of these are the treatments of *Salix* (Shlyakov 1954) and *Hippuris* (Semenova-Tian-Shanskaya 1959), descriptions of new species of *Cotoneaster* and *Anthyllis* (Yuzepchuk 1950), *Sonchus* (Orlova 1964) etc. Yuzepchuk included the treatments of the Murmansk material of *Alchemilla* (Yuzepchuk 1954) and *Euphrasia* (Yuzepchuk 1955a) into a broader context. Some specimens collected during these revisions were distributed in the exsiccata published by the Komarov Botanical Institute (e.g. Yuzepchuk 1955b). Despite the opportunity to publish separate background papers, many species established as new to science in volumes 3–5 were described in appendices added to the main text in these books.

## Temporal coverage

**Formation period:** 1837–1965.

## Usage licence

### Usage licence

Other

### IP rights notes

Creative Commons Attribution (CC-BY) 4.0 License

## Data resources

### Data package title

Distribution of vascular plants in Murmansk Region (Russia) as represented in the *Flora of Murmansk Region* (1953–1966)

### Resource link

https://www.gbif.org/dataset/f38b3f41-cd27-4e8e-92b9-7f1ecb47e05a

### Alternative identifiers

https://doi.org/10.15468/ub7xkx

### Number of data sets

1

### Data set 1.

#### Data set name

*Flora of Murmansk Region* (1953–1966) point distribution data

#### Number of columns

22

#### Description

The occurrence of vascular plant species published on species distribution maps in the *Flora of Murmansk Region* (1953–1966).

**Data set 1.** 

Column label	Column description
occurrenceID	An identifier for the occurrence (unique).
basisOfRecord	The specific nature of the data record.
taxonRemarks	Comments or notes about the taxon or name [name as in the *Flora of Murmansk Region*].
scientificName	The binary scientific name (species name), without authorship and date information, or hybrid formula (for interspecific hybrids).
taxonRank	The taxonomic rank of the mapped taxon, corresponding to the scientificName.
genus	The full scientific name of the genus in which the taxon is classified.
specificEpithet	The name of the first or species epithet of the scientificName.
family	The full scientific name of the family in which the taxon is classified.
eventDate	The interval during which the original data were obtained (herbarium specimens were collected).
decimalLatitude	The geographic latitude (in decimal degrees, using the spatial reference system given in geodeticDatum) of the geographic centre of a Location. Positive values are north of the Equator, negative values are south of it. Legal values lie between -90 and 90, inclusive.
decimalLongitude	The geographic longitude (in decimal degrees, using the spatial reference system given in geodeticDatum) of the geographic centre of a Location. Positive values are east of the Greenwich Meridian, negative values are west of it. Legal values lie between -180 and 180, inclusive.
geodeticDatum	An EPSG code of the Spatial Reference System (SRS) [WGS 84, used consistently].
coordinateUncertaintyInMeters	The horizontal distance (in metres) from the given decimalLatitude and decimalLongitude describing the smallest circle containing the whole of the Location.
georeferencedBy	A name of the person who determined the georeference (spatial representation) for the Location [Chernov, Evgeny Georgievich].
countryCode	The standard code for the country in which the Location occurs [RU, Russia].
stateProvince	The name of the next smaller administrative region than country (state, province, canton, department, region etc.) in which the Location occurs [Murmansk Oblast].
license	A legal document giving official permission to do something with the resource [Creative Commons Attribution (CC-BY) 4.0 License].
institutionID	An identifier for the institution having custody of the object(s) or information referred to in the record [Polar-Alpine Botanical Garden-Institute]
institutionCode	The name (or acronym) in use by the institution having custody of the object(s) or information referred to in the record [KPABG].
bibliographicCitation	A bibliographic reference for the resource [*Flora of Murmansk Region*] as a statement indicating how this record should be cited (attributed) when used.
DatasetName	The name identifying the data set from which the record was derived.
language	A language of the resource.

## Supplementary Material

FE93C29A-8B45-5100-B609-E52A68992394 10.3897/BDJ.8.e59456.suppl1 Supplementary material 1 The occurrence of vascular plant species published on species distribution maps in the Flora of Murmansk Region (1953–1966) Data type occurrences File: oo_461561.txt https://binary.pensoft.net/file/461561 Kozhin M.N., Lommi S., Sennikov A.N.
